# Inhaled nebulised medications in palliative care - a survey among palliative care practitioners in Germany

**DOI:** 10.1186/s12904-025-01761-y

**Published:** 2025-05-04

**Authors:** Ruth Mair, Annette Schnell, Christine Steger-Arand, Wolfgang Herr, Michael Rechenmacher

**Affiliations:** 1 Department of Internal Medicine III, University Hospital Regensburg, Regensburg, Germany; 2 Centre for Palliative Care, University Hospital Regensburg, Regensburg, Germany

**Keywords:** Palliative care, Inhalation, Inhalative, Nebulisation, Nebulised, Symptom control

## Abstract

**Background:**

In palliative care, alternative routes for drug application besides the oral and intravenous administration are frequently necessary. Up-to-date, very little is known about the familiarity, use and perceived relevance of inhalative medications for symptom control among palliative care practitioners.

**Methods:**

We conducted an anonymous online survey among palliative care physicians throughout Germany between 09/2021 and 04/2022. The questionnaire covered participants’ sociodemographics, as well as familiarity, perceived relevance and prescription practices regarding 21 nebulised drugs. Analysis was performed using methods of descriptive statistics.

**Results:**

108 fully completed questionnaires were analysed. Most of the participants were employed in palliative care for 5 + years. The administration of normal saline, mucoactive drugs, bronchodilators and steroids via nebulisation was a widely known and frequently used technique among the participants, as evidenced by its regular use in clinical routine. About 50% of the participants reported to know epinephrine and tranexamic acid for anti-oedematous or haemostyptic effects, respectively. Both drugs were considered “relevant” by more than 60% of the prescribers. Only a minority of participants reported to know and use nebulised opioids, iloprost, several antibiotics, heparin, ketamine and lidocaine.

**Conclusions:**

Our survey shows that nebulised drugs are prescribed and considered relevant in palliative care. However, for several of the mentioned medications only limited data is available regarding use and effectivity. There is also uncertainty to what extent the existing data may be transferable into routine palliative care setting. Therefore, more evidence should be generated.

**Trial registration:**

Not applicable.

**Supplementary Information:**

The online version contains supplementary material available at 10.1186/s12904-025-01761-y

## Background

A variety of problems can occur at the end of life and the treatment of symptoms like pain, dyspnoea or bleeding can be challenging [1]. Many drugs that are regularly administered to control these issues are available via the oral, transdermal or intravenous route. Furthermore, in palliative care there is a wide approval for subcutaneous drug administration, mostly in form of a so-called “off-label-use” based upon expert opinion [2]. Another way of application is inhalation of nebulised drugs. There are several reasons, why to consider the inhalation of medications in palliative care. First, there may be situations, where an oral intake is not possible anymore, for example due to dysphagia, and at the same time a parenteral application may not be available or tolerated by the patient. It might also be taken into consideration, that inhalation could offer several advantages over other routes of administration. These comprise direct delivery to the bronchial system and therefore higher luminal doses, less systemic side effects and a faster onset of action [3].

For some medications, the inhalative use is approved and well established in daily clinical routine. These include normal saline, steroids and bronchodilators [4, 5]. There are also a variety of trials published on the inhalative use of other medications. However, for most of these medications evidence for the nebulised route of administration is altogether very limited. Furthermore, most of the published data on inhalation of the various drugs were not collected in palliative care situations.

To date, there is very little known about the knowledge, use and perceived relevance of inhalative medications for symptom control among palliative care practitioners. Therefore, this survey aimed at investigating this unexplored topic of inhalation therapies for symptom control in the palliative care setting from the view of palliative care professionals in Germany.

## Methods

### Study design

We conducted an online survey to evaluate the role of inhalation therapies for symptom control in the palliative setting from the view of physicians in palliative care in Germany. For this purpose, palliative care units, specialised palliative home care (SAPV)-teams, as well as other palliative care physicians throughout Germany were invited to participate by email. In an attempt to increase the study population, participants were asked to forward the survey invitation to any other palliative care professionals.

Invitations were sent on September 9, 2021. Data from questionnaires completed until April 21, 2022 were included into the present analysis.

The survey was designed to be carried out in a fully anonymous way. An identification of individual respondents by the research team was not possible.

The study was discussed with the local Ethics Committee. The need for a formal approval of the study was waived, due to completely anonymous participation.

### Data collection and questionnaire

Data collection was done via a commercial online survey platform (UmfrageOnline, enuvo GmbH, Switzerland).

The questionnaire covered participants’ sociodemographics in addition to familiarity and prescription practices regarding nebulised medications.

Regarding participant´s characteristics, items on age, gender, palliative care qualification, profession and occupational specifics were included.

Medications suitable for inhalation in a palliative care setting were identified on the basis of expert knowledge and via literature research. Finally, 21 drugs were selected for the survey. The following variables were assessed for each drug: familiarity with the specific nebulised drug in the palliative care setting (yes/no), prescription in palliative care clinical routine (yes/no), frequency of prescription (very often/often/less often/rarely/never), indication for prescription in palliative care (drug-specific categories), opinion regarding clinical relevance for symptom relief (very relevant, relevant, less relevant, not relevant at all), clinical setting of prescription (specialised palliative home care, palliative care unit, palliative care inpatient consultation, non-palliative care setting), dosing (drug-specific categories), administration interval (drug-specific categories), and combination with other inhalative medications (yes/no and free text reply). If a participant did not know a medication or if he answered that he does not prescribe a medication, no further questions appeared concerning this specific drug.

### Statistical analysis

Analysis was performed using methods of descriptive statistics. Individual items of the questionnaire were reported with absolute numbers and/or as percentages. Particularly relevant aspects were presented by bar charts.

Only complete questionnaires were analysed.

Statistical analysis was performed with R (R Foundation for Statistical Computing, Vienna 2021).

## Results

### Baseline characteristics of participants

1328 email addresses were contacted (comprising institutions and individual persons). 188 persons participated in the survey. 128 of these completed the questionnaire. The survey was primarily answered by physicians; only very few questionnaires were answered by nursing staff and non-medical palliative care staff. Furthermore only doctors are allowed to prescribe drugs. Therefore, nursing staff and non-medical palliative care staff were excluded from the analysis. Finally, there were 108 questionnaires considered for the analysis.

Details concerning the participant characteristics are shown in Table 1. 58% were female. Most participants were between 40 and 59 years old. The majority held a certificate of palliative care qualification and worked in the field of specialised palliative care. About half of the participants had been working in palliative care for at least 10 years, only 5% had less than one year of experience. More than 50% of the participants worked in a hospital. Therefore, the participants reflect the inpatient as well as the outpatient sector of specialised palliative care physicians.

**Table 1:** Participants’ characteristics

Variable	Distribution
**Gender**	**n (%)**
Male	45 (42)
Female	62 (58)
Missing	1 (-)
**Age**	**n (%)**
≤ 40 years	13 (12)
40–49 years	29 (27)
50–59 years	49 (45)
60–69 years	14 (13)
≥ 70 years	3 (3)
missing	0 (-)
**Certificate of palliative care qualification**	**n (%)**
Yes	101 (94)
No	6 (6)
Missing	1 (-)
**Work in specialised ** **palliative care**	**n (%)**
Yes	101 (94)
No	7 (6)
Missing	0 (-)
**Professional experience in specialised ** **palliative care**	**n (%)**
< 1 year	5 (5)
1–4 years	20 (20)
5–9 years	23 (23)
≥ 10 years	52 (52)
missing	8 (-)
**Work setting**	**n (%)**
Hospital	60 (56)
Doctor’s office	17 (16)
Retired	0 (0)
Other	24 (22)
Hospital/ doctor’s office/ retired **+** other	6 (6)
Missing	1 (-)
**Position**	**n (%)**
Chief/leading physician (Leitende Position/Chefarzt/Chefärztin)	29 (27)
Senior physician (Oberarzt)	34 (31)
Medical specialist (Facharzt)	39 (36)
Assistant doctor (Assistenzarzt)	2 (2)
General practitioner (Allgemeinarzt)	4 (4)
Missing	0 (-)

### Familiarity with inhalative drugs

**Fig. 1:**
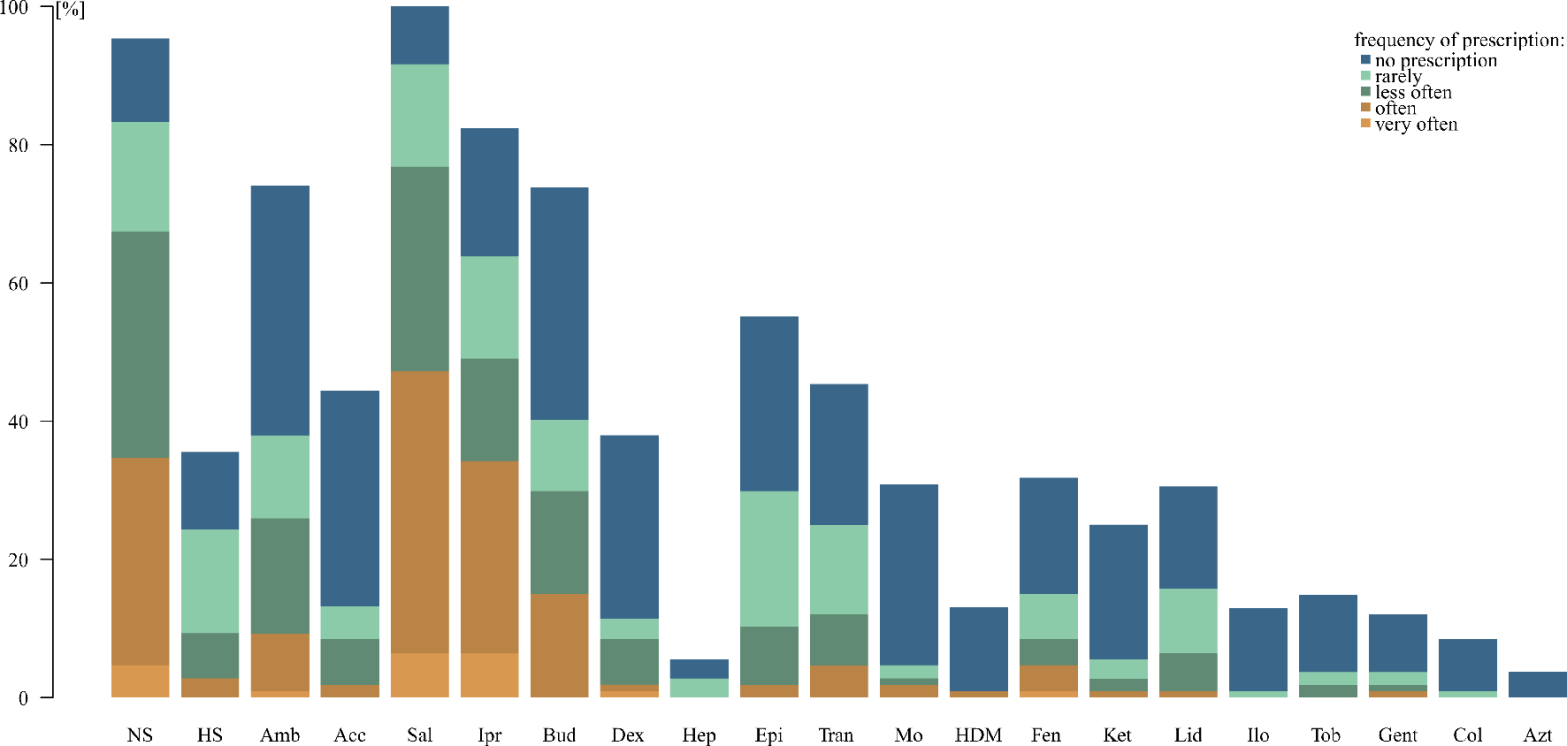
Familiarity regarding nebulised drugs and frequency of their prescription. legend: Total bar height = proportion of participants who are familiar with a specific nebulised drug (percentage). Blue bar = proportion of participants who are familiar with a specific nebulised drug, but do not prescribe it. Brown and green bars = proportion of participants who are familiar with a specific nebulised drug and prescribe it “very often” (light brown), “often” (dark brown), “less often” (dark green) or “rarely” (light green). NS = normal saline, HS = hypertonic saline, Amb = ambroxol, Acc = acetyl cysteine, Sal = salbutamol, Ipr = ipratropium bromide, Bud = budesonide, Dex = dexpanthenol, Hep = heparin, Epi = epinephrine, Tran = tranexamic acid, Mo = morphine, HDM = hydromorphone, Fen = fentanyl, Ket = ketamine, Lid = lidocaine, Ilo = iloprost, Tob = tobramycin, Gent = gentamicin, Col = colistin, Azt = aztreonam

The proportion of participants that reported being familiar with each drug is shown in Fig. 1, indicated by the total bar height.

Almost all participants were familiar with the inhalative application of normal saline 0.9%, a drug for the hydration of mucous membranes. With regard to mucoactive substances, nebulised ambroxol was far better known than hypertonic saline or acetylcysteine.

Inhalation of the bronchodilators salbutamol and ipratropium bromide was known by a majority, as well as the inhalation of the steroid budesonide. In terms of other anti-inflammatory drugs, inhalation of dexpanthenol was known by about a third of participants, whereas nebulisation of heparin as an anti-inflammatory drug was almost unknown.

About half of the participants were familiar with nebulisation of the anti-oedematous and vasoconstrictive drug epinephrine and of haemostyptic tranexamic acid.

Regarding opioids, which are used to treat pain, dyspnoea and cough, less than one third of participants were familiar with the inhalation of morphine, hydromorphone or fentanyl. Inhalation of the two other analgesic drugs assessed, ketamine and lidocaine, was also scarcely known.

Iloprost is a drug for treatment of pulmonary hypertension. Only very few participants were familiar with inhalation of this drug. The same was true for the nebulisation of the antibiotics tobramycin, gentamicin, colistin and aztreonam.

In summary, only five inhalative drugs were known by more than half of the participants: normal saline 0.9%, ambroxol, salbutamol, ipratropium bromide, budesonide and epinephrine. All other nebulised drugs were known to a lower extent.

### Clinical use

The frequency of clinical use, as reported by the physicians for each medication they were familiar with, varied widely by drug (Fig. 1).

For example, concerning the well-known inhalative drugs normal saline 0.9%, salbutamol and ipratropium bromide, the prevalence of any prescription was higher than 75%. About half of the prescribers reported a frequent or very frequent prescription. Data for budesonide were similar but the prevalence was a bit lower.

Concerning epinephrine and tranexamic acid about 50% reported prescription of the drugs, too. However, the drugs were prescribed “very often” or “often” by only 6% and 19% respectively.

For other substances, like acetylcysteine or dexpanthenol, less than half of the practitioners reported any prescription. The proportion of participants reporting frequent prescription of these drugs was very low.

For less well-known nebulised substances the prevalence of prescription was generally low. The absolute number in these cases was very small.

### Indication for use in palliative care, dosing, administration interval, and combination with other medications

The reported indications for treatment with the various nebulised drugs in the palliative care setting are shown in Table 2.

**Table 2:** Reported indications for the prescription of the various drugs

	number of pre-scribers	expectorant/ mucolytic	hydration of mucous membranes	broncho-dilation	anti- inflammatory, oral	anti-inflammatory, hypopharyngeal	anti-inflammatory, tracheal	anti-inflammatory, pulmonary	free text reply
Normal saline 0.9%	90	80/90	73/90	-	-	-	-	-	(dry) cough, dyspnoea, prophylaxis, structuring of the patient’s day, well-being, various lung diseases, diluent
Hypertonic saline	26	26/26	-	-	-	-	-	-	cough inducing
Ambroxol	41	41/41	-	-	-	-	-	-	on patient’s request
Acetyl cysteine	14	14/14	-	-	-	-	-	-	
Salbutamol	99	-	-	97/98	-	-	-	-	dyspnoea, secretolysis, spasmolysis, well-being
Ipratropium bromide	69	-	-	68/68	-	-	-	-	chronic obstructive pulmonary disease, asthma, sialorrhea
Budesonide	43	-	-	30/43	-	23/43	-	23/43	cough, respiratory congestion, anti-inflammatory in general (nasopharyngeal, tracheal, pulmonary)
Dexpanthenol	12	-	-	-	9/12	9/12	8/12	8/12	cough, epistaxis, mucous membrane care
Heparin	3	-	-	-	-	-	-	2/2	
	**number of pre-scribers**	**anti-oedematous**	**haemostyptic**,** oral**	**haemostyptic**,** hypo-pharyngeal**	**haemostyptic**,** pulmonary**	**dyspnoea**	**cough**	**pain**	**free text reply**
Epinephrine	32	26/31	-	-	-	-	-	-	bleeding (*n* = 15); bronchodilation
Tranexamic acid	27	-	13/26	20/26	23/26	-	-	-	haemostyptic nasal
Morphine	5	-	-	-	-	4/5	1/5	5/5	
Hydromorphone	1	-	-	-	-	1/1	0/1	1/1	
Fentanyl	16	-	-	-	-	12/16	3/16	16/16	
Ketamine	6	-	-	-	-	-	-	5/5	
Lidocaine	17	-	-	-	-	-	12/17	11/17	dry cough
	**number of pre-scribers**	**dyspnoea (pulmonary hypertension)**	**acute pneumonia**	**cystic fibrosis**	**suppressive therapy of chronic pulmonary infection**				**free text reply**
Iloprost	1	1/1	-	-	-	-	-	-	
Tobramycin	4	-	0/4	3/4	4/4	-	-	-	
Gentamicin	4	-	0/4	2/4	4/4	-	-	-	
Colistin	1	-	0/1	1/1	1/1	-	-	-	
Aztreonam	0	-	0/0	0/0	0/0	-	-	-	

Multiple answers were possible. “-“: response option was not available for the respective drug. Number of prescribers: number of participants who reported prescription of the specific drug for any indication

Numbers indicate “the number of participants who reported prescription of a drug for the respective indication”/ ”the number of participants who answered the question regarding the indication for prescription of this specific drug”

Answers regarding dosing and administration intervals revealed a large variability in prescription practices. The combination with other inhalative drugs likewise varied widely. This resulted in many reply categories with mostly very small numbers. Details are shown in the additional file 1, together with data on the clinical setting of use.

### Clinical relevance

**Fig. 2:**
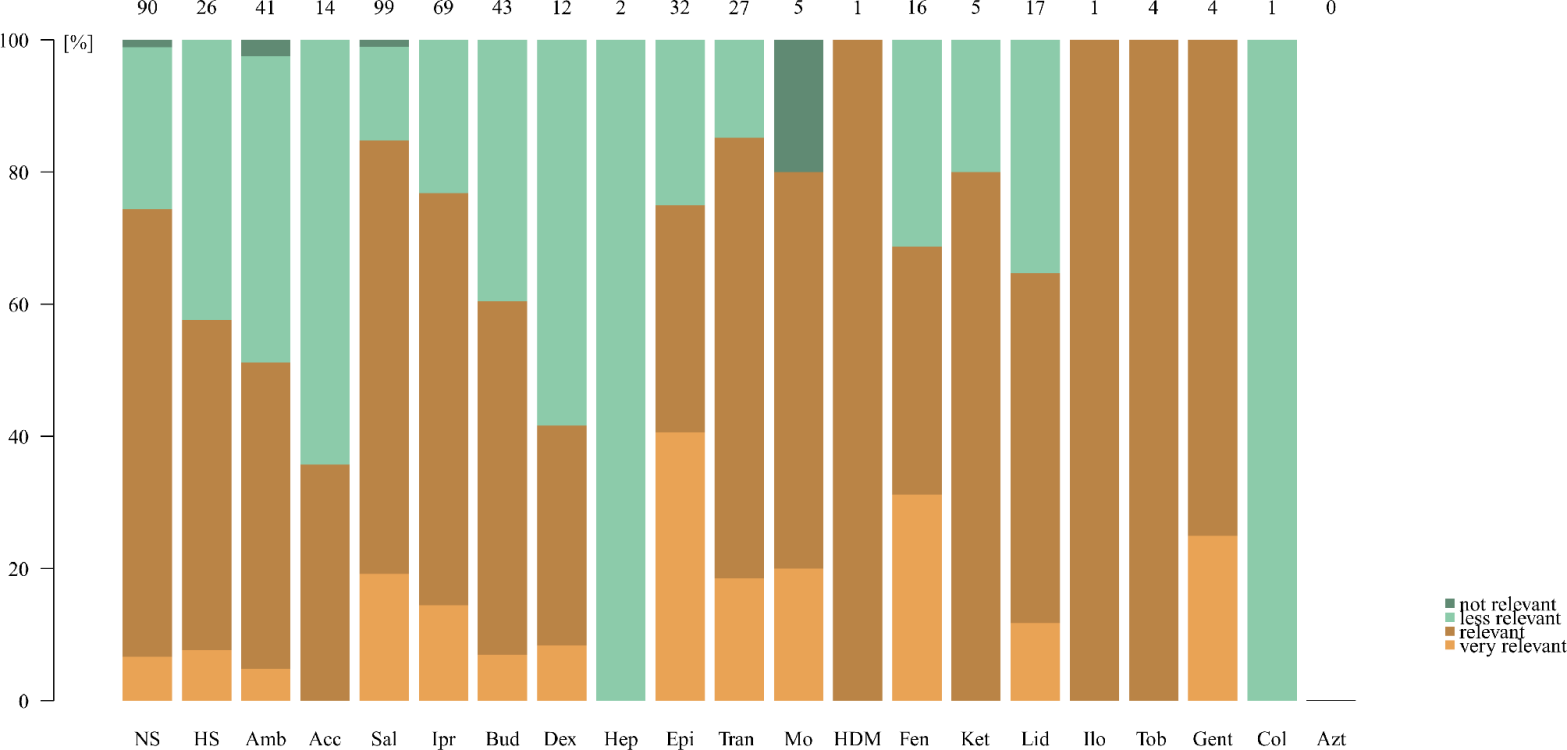
Perceived relevance regarding nebulised drugs. legend: Brown and green bars = proportion of prescribers, who deem the relevance of a specific nebulised drug “very relevant” (light brown), “relevant” (dark brown), “less relevant” (light green) or “not relevant” (dark green). The absolute number of participants reporting to prescribe a specific drug is shown above each bar. NS = normal saline, HS = hypertonic saline, Amb = ambroxol, Acc = acetyl cysteine, Sal = salbutamol, Ipr = ipratropium bromide, Bud = budesonide, Dex = dexpanthenol, Hep = heparin, Epi = epinephrine, Tran = tranexamic acid, Mo = morphine, HDM = hydromorphone, Fen = fentanyl, Ket = ketamine, Lid = lidocaine, Ilo = iloprost, Tob = tobramycin, Gent = gentamicin, Col = colistin, Azt = aztreonam

Opinions regarding the clinical relevance by practitioners reporting the use of nebulised drugs varied widely by substance (Fig. 2).

More than 60% of the prescribers considered the nebulised bronchodilators salbutamol and ipratropium bromide, as well as the nebulised steroid budesonide, to be “relevant” or “very relevant” in clinical use. The same was true for epinephrine and tranexamic acid.

Nebulised normal saline 0.9% was also estimated as a relevant drug by a major percentage of participants. The relevance of the mucoactive drugs hypertonic saline, ambroxol and acetylcysteine was deemed lower, with more participants considering hypertonic saline and ambroxol relevant than acetylcysteine.

Concerning all other drugs, the absolute numbers were low. Nevertheless, among the few prescribers of these drugs, many considered them to be relevant.

## Discussion

Our study among palliative care professionals in Germany sheds first light on the clinical use and perceived relevance of inhalative medications for symptom control in the palliative care setting. Our data revealed that inhaled drugs are regularly prescribed and often considered relevant in palliative care. Nevertheless, there were substantial differences between distinct substances. Participants reported familiarity and frequent use especially for those drugs, whose nebulised use is generally approved and well established in clinical routine. Inhalation of other drugs was almost unknown, for example inhalation of nebulised heparin or antibiotics.

There are several barriers for desired studies on nebulised medications in palliative care [6]. Published studies concerning non-approved inhaled drugs are mostly very small controlled trials or uncontrolled studies such as case reports [7, 8]. Most of the studies were performed in non-palliative care populations and therefore it remains unclear, to what extent the results can be transferred to a palliative care setting. Nevertheless, they may provide some valuable information - especially for palliative care situations, where no approved medication exists or no approved way of application is available for symptom control.

### Normal saline 0.9% and mucoactive substances hypertonic saline, ambroxol and acetylcysteine

Nebulised normal saline 0.9% is widely known in in the medical field. By indication, it is applied for the hydration of mucous membranes; however, administration of normal saline is also commonly intended as mucoactive therapy in daily clinical practice. This was also reflected by the results of the present survey. Most participants reported knowing and prescribing the nebulised form of this drug. Intriguingly, beyond the standard indications, some participants reported to use it for “well-being” or for “structuring of the patient´s day”. This might be interpreted as normal saline sometimes being used as an intervention for the sake of doing something– therefore being considered as a rather psychological substance with limited pharmaceutical activity, but also virtually free of side effects.

Hypertonic saline, ambroxol and acetylcysteine are regarded as mucoactive therapies. The intended mechanism of mucoactive drugs is to affect mucous properties and to improve the clearance of secretion. Besides others they act as expectorants, mucolytics, mucokinetics or abhesives [9, 10]. The effect of mucoactive drugs is discussed widely but controversially [10, 11]. Concerning the inhalative use of mucoactive agents, most of the published work studied the effect in cystic fibrosis and paediatric acute bronchitis [12–14]. Hypersecretion and difficulties with mucous clearance are common in palliative care patients. However, the pathophysiology of symptoms in adult palliative care patients might be very different compared to paediatric bronchitis or cystic fibrosis. Thus, the aforementioned evidence may not be applicable to our patient collective. A more comparable condition might be non-cystic fibrosis. However, available data concerning the clinical effects in this setting are also ambiguous [15]. We did not find any published data regarding the inhalative use of mucoactive agents in a clearly palliative situation. Nevertheless our data reveal that a certain proportion of our study participants consider nebulised mucoactive substances as relevant medications and up to a third of the participants prescribe them in palliative care situations.

### Bronchodilators and Budesonide, other anti-inflammatory substances

#### Bronchodilators and Budesonide

The nebulised bronchodilators salbutamol, ipratropium bromide and budesonide are very well established in daily clinical practice. Their efficacy has been extensively investigated, and major guidelines concerning chronic obstructive pulmonary disease (COPD) and asthma recommend their use [4, 5]. Therefore, it is not surprising, that a large percentage of this survey´s participants prescribe these drugs for bronchodilation also in the palliative care setting.

#### Dexpanthenol

Dexpanthenol is a drug that is commonly used for its anti-inflammatory and wound-healing effects [16]. In our clinical experience, the nebulisation of dexpanthenol is often recommended for tracheitis by expert opinion. However, there are no official recommendations and no studies or case reports analysing inhaled dexpanthenol could be found. Nevertheless, about two thirds of the participants were familiar with its inhalation and about one third of them reported to prescribe it. Likewise one third of participants considered it as a relevant drug.

#### Heparin

Heparin is mainly known for its anticoagulant properties. Furthermore, anti-inflammatory, anti-microbial and mucoactive effects have been described in the literature [17]. The results of our survey suggest, that it is used only very rarely as a nebulised drug in the palliative care setting. We could not find any studies reporting on the use of inhaled heparin in a dedicated palliative care situation. The focus of published clinical studies is mainly on acute lung injury in critically ill ventilated patients, especially in acute smoke inhalation injury, COPD and asthma [18–20]. Positive effects on different parameters like bronchoconstriction, lung function parameters, ventilator free days, length of hospital stay and mortality rates have been suggested. However, data are controversial and endpoints varied widely [19–21].

Given the rather special nature of the various study populations, it remains unclear if and to what extent inhaled heparin could exert beneficial effects in palliative care patients. One pilot study in non-ventilated COPD patients, which may be somewhat more transferable to our setting, described an improvement not only in lung function parameters, but also in the clinically relevant end point of dyspnoea [8]. With respect to bleeding complications, the evidence seems to support, that there is no increased risk of bleeding [19]. However, this might also differ in our palliative care patients, especially when suffering from cancer affecting the lungs.

### Bleeding control (and bronchodilation) by epinephrine and Tranexamic acid

#### Tranexamic acid

Tranexamic acid rewards special attention as a potential option for bleeding control. It is an antifibrinolytic agent, which is frequently administered systemically to stop bleeding [22]. Some studies point towards the effectiveness of systemic tranexamic acid also in hemoptysis, but the effect is not yet certain [23]. Meanwhile there is a growing number of mostly small studies and case reports, which report on the successful use of nebulised tranexamic acid for bleeding control [7, 24]. Particularly, a randomised controlled study including 47 patients showed a benefit of inhaled tranexamic acid for the termination of non-massive pulmonary bleeding [25]. In addition, case reports are published using inhaled tranexamic acid for post-tonsillectomy bleeding and epistaxis [26, 27]. There were no severe side effects reported for the use in either indication [7, 24, 25]. More of the participants of our survey reported on using it for pulmonary bleeding, than for oral or hypopharyngeal bleeding. Interestingly, most of them deemed it a relevant inhalative drug for bleeding events.

#### Epinephrine

The participants of our survey also considered epinephrine as a relevant nebulised drug. Furthermore, a considerable amount of participants reported to also administer epinephrine for the termination of bleeding events. The approval of nebulised epinephrine, however, encompasses only mucosal oedema of the upper airway and/ or bronchoconstriction, caused by conditions like laryngotracheitis or allergic reactions [28]. Nonetheless, epinephrine has vasoconstrictive effects and therefore can provide haemostasis. Topical epinephrine is used for the management of bleeding, for example during bronchoscopy or oral surgery [29, 30]. Evidence for the nebulised administration route for haemostasis is much more limited, but the successful use of nebulised epinephrine for termination of oral bleeding has been reported [31].

### Opioids, other analgesic drugs

#### Opioids

Opioids are important medications for the treatment of pain, dyspnoea and cough in the palliative care setting [1]. Besides the oral and intravenous application, opioids are often administered subcutaneously. In addition, Fentanyl is available as nasal spray, transdermal patch and dispersible tablet. The existence of multiple, easily applicable administration routes might be one reason for the very low frequency regarding the use of inhaled opioids as reported in our study. The slightly higher reported use of inhaled fentanyl might be overestimated, since free text answers suggested, that not all participants distinguished between inhalation and intranasal application.

A further potential explanation for the low frequency of inhalation is the more limited available evidence regarding the nebulised route of administration compared to the established modes of application. Most published studies on inhaled opioids refer to morphine, only some refer to fentanyl. Only very limited data exist on inhaled hydromorphone [32, 33].

Regarding the treatment of pain, most of the few small prospective studies suggest a benefit for nebulised morphine, although the administered doses of application varied [34–36]. Besides, smaller studies point towards a benefit of inhaled fentanyl for relieve of pain in a postoperative and emergency setting [37].

Recent meta-analyses questioned the effect of opioids on dyspnoea at all [38, 39]. Most of the meta-analyses and systematic reviews revealed a lack of evidence for the benefit of inhaled morphine for objective and subjective parameters in various diseases as well [40–42]. Even though non randomised controlled studies and case reports might suggest a benefit, nebulised fentanyl also did not show significant effects on dyspnoea in randomised controlled studies [43–46].

Concerning the treatment of coughing there are only a few case reports, which described an improvement after inhalation of morphine [47].

#### Ketamine

We found a few case reports and some small randomised studies pointing towards an analgesic effect of nebulised ketamine in acute pain in emergency settings and for the management of the postoperative sore throat [48, 49]. One study reported on the use of inhaled ketamine in corticosteroid resistant asthma exacerbation [50]. Only very few participants of our survey stated to use inhaled ketamine for the treatment of pain. Nevertheless, there are a number of potential applications for patients in (specialised) palliative care, for example, when complex out-patient pain therapy is required in situations where parenteral administration is not desired or possible.

#### Lidocaine

A few reports have been published on the use of nebulised lidocaine for the treatment of cough due to various conditions, as for example COPD, infection or cancer [51–53]. Its inhalation was discussed as an alternative option, when other antitussive medication was not successful or tolerable [52]. There is also limited evidence for the use of nebulised lidocaine for treatment of pain, with one analysis showing a reduction of pain during nasogastric tube insertion when lidocaine was nebulised prior to the intervention [54]. This might suggest a use in palliative care patients who need additional local analgesic - for example due to mucositis or an oropharyngeal tumour - however not being able to rinse their mouth with a local anaesthetic solution. The use of inhaled lidocaine seems to play only a small role in palliative care, since only about a quarter of the study participants reported to know it and only about half of them reported to (rarely) use it.

### Iloprost

Inhaled iloprost is a drug for the treatment of pulmonary hypertension [55]. This potentially terminal illness may obviously also be encountered in the palliative care setting. With respect to our data, it did not play a relevant role. Very few participants were familiar with this drug and almost nobody reported to use it. This could be different in patient populations enriched with pulmonary pathology.

### Antibiotics

Almost no participants of our survey reported to know or to prescribe the nebulised antibiotics tobramycin, gentamicin, colistin and aztreonam. These antibiotics act on Gram-negative organisms, including *Pseudomonas aeruginosa* [56]. Inhaled antibiotics were primarily tested in cystic fibrosis, and limited data are also available for non-cystic-fibrosis and ventilator associated pneumonia [56, 57]. Besides this, they are often reserved for the use in multi-drug-resistant bacterial infections, but resistances have already been described [58]. Therefore, the indication for administration of nebulised antibiotics should also be handled very consciously and cautiously in the palliative care setting.

### Limitations

Some limitations should necessarily be kept in mind when considering our results. Because of the type of the study and the mode of the invitation process, we cannot be sure to what extent the results are transferable to the palliative care community in general. In addition, especially for the less known inhalative drugs the absolute numbers for answers concerning details of use were small. Therefore, any interpretation should be done with caution. However, given the aforementioned paucity of prior information, our study provides valuable first insight into the clinical relevance of this topic.

## Conclusions

To our knowledge, this is the first study on the use and perceived relevance of inhaled medications for symptom control among palliative care physicians in Germany. Our study revealed that nebulised drugs are a standard component of clinical palliative care and are frequently regarded as a valuable therapeutic option by the participants of the survey. Concurrently, our analysis of the findings reveal a paucity of published data concerning the specific utilisation and efficacy of nebulised medications in palliative care. For the majority of the substances, the scientific evidence for this route of administration exists in non-palliative settings. However, analogous conclusions can be drawn for some areas of palliative care, although the results may not be directly transferable to palliative care patients. Overall, even though there are several barriers to trials on nebulised medications, our study encourages further research to optimise symptom control in palliative care using the inhaled approach.

## Electronic supplementary material

Below is the link to the electronic supplementary material.

Supplementary Material 1

## Data Availability

Data and materials are available from the corresponding author upon reasonable request.

## Abbreviations

COPD: Chronic obstructive pulmonary disease
SAPV: Specialised palliative home care
