# Supplementary material for: Inhaled nebulised medications in palliative care - a survey among palliative care practitioners in Germany
Source: BMC Palliat Care. 2025 May 4;24:125. doi: 10.1186/s12904-025-01761-y (PMC12051283; doi:10.1186/s12904-025-01761-y)
Supplement: Supplementary file 1 — Supplementary Material 1 [file 12904_2025_1761_MOESM1_ESM.pdf]

Supplementary material

Data for each drug concerning:

- administration interval (drug-specific categories and free text reply)
- dosing (drug-specific categories and free text reply)
- combination with other inhalative medications (yes/no and free text reply)
- clinical setting of prescription (palliative care ward, specialized palliative home care, palliative care inpatient consultation, non-palliative care setting)

Abbreviations:
VOR (various other replies not indicating a specific administration interval/dosing)
NA (not applicable/not available)
PCU (palliative care unit)
SAPV (specialized palliative home care)
PCIC (palliative care inpatient consultation)
NP (non-palliative care setting)
HS (hypertonic saline)
FT (free text reply)
IU (international units)
PRN (pro re nata, if the situation requires medication)

**Normal saline 0,9%**									
administration interval	n	dosing		combination		combination with	setting	n
1 x / day	1	NA		NA		NA	PCU	49
2 x / day	8						SAPV	44
3 x / day	48						PCIC	30
at least 3 x / day	1						NP	34
3-4 x / day	2						NA	0
4 x / day	24							
VOR	6							
NA	0							

**Hypertonic saline**								
administration interval	n	dosing	n	combination		combination with	setting	n
1 x / day	3	3%	22	NA		NA	PCU	10
2 x / day	10	6%	4				SAPV	18
3 x / day	12	7%	4				PCIC	6
4 x / day	1	NA	0				NP	4
NA	0						NA	0

Ambroxol								
administration interval	n	dosing	n	combination	n	combination with	setting	n
1 x / day	4	15 mg	38	yes	17	HS	PCU	26
2 x / day	35	22.5 mg	3	no	24	HS - salbutamol - ipratropium bromide	SAPV	17
up to 4 x / day	1	NA	0	NA	0	salbutamol - ipratropium bromide	PCIC	13
VOR	1						NP	10
NA	0						NA	0

Acetylcysteine								
administration interval	n	dosing	n	combination	n	combination with	setting	n
1 x / day	8	300 mg (3ml)	13	yes	6	HS	PCU	7
2 x / day	5	FT: 600 mg BT	1	no	8		SAPV	7
VOR	1	NA	0	NA	0		PCIC	3
NA	0						NP	4
							NA	0

Salbutamol								
administration interval	n	dosing	n	combination	n	combination with	setting	n
1 x / day	2	1.25 mg (5 drops)	45	yes	73	HS	PCU	53
2 x / day	15	2.5 mg (10 drops)	60	no	26	ipratropium bromide	SAPV	46
2 - 3 x /day	2	FT: 1 mg (4 drops)	1	NA	0	HS - ipratropium bromide	PCIC	35
2 - 4 x / day	1	FT: 2 mg (8 drops)	1			ipratropium bromide - Budesonid	NP	39
up to 3 x / day	1	VOR	4			HS - ipratropium bromide - budesonide	NA	0
3 x / day	53	NA	1					
3 - 4 x / day	1							
up to 4 x / day	1							
4 x / day	17							
3 - 6 x / day	1							
up to 6 x / day	1							
PRN	1							
VOR	3							
NA	0							

**Ipratropium bromide**									
**administration interval**	n	**dosing**	n	**combination**	n	**combination with**	**setting**	n
1 x / day	5	250 µg	48	yes	48	HS	PCU	35
2 x / day	13	500 µg	12	no	21	salbutamol	SAPV	34
3 x / day	35	FT: 125-250 µg	1	NA	0	HS - salbutamol	PCIC	21
3 - 4 x/ day	1	VOR	9			salbutamol - budesonide	NP	26
3-6 x / day	1	NA	2			metered dose inhaler: fenoterol	NA	0
up to 4 x / day	1							
4 x / day	10							
PRN	1							
VOR	2							
NA	0							

**Budesonide**								
**administration interval**	n	**dosing**	n	**combination**	n	**combination with**	**setting**	n
1 x / day	8	0.5 mg	14	yes	6	salbutamol - ipratropium bromide	PCU	25
1 - 2 x / day	1	1 mg	27	no	37		SAPV	20
2 x / day	23	2 mg	6	NA	0		PCIC	16
3 x / day	6	FT: 0.2 - 0.4 mg	1				NP	19
4 x / day	4	VOR	3				NA	0
NA	1	NA	2					

**Dexpanthenol**								
**administration interval**	n	**dosing**	n	**combination**	n	**combination with**	**setting**	n
2 x / day	2	50 mg	6	yes	6	HS	PCU	4
3 x / day	7	100 mg	4	no	6	xylometazoline	SAPV	7
4 x / day	1	NA	2	NA	0		PCIC	4
up to 6 x / day	1						NP	5
VOR	1						NA	0
NA	0							

**Heparin**								
**administration interval**	n	**dosing**	n	**combination**	n	**combination with**	**setting**	n
1 x / day	1	25000 IU	3	yes	1	HS	PCU	1
2 x / day	2	NA	0	no	2		SAPV	2
NA	0			NA	0		PCIC	0
							NP	1
							NA	0

Epinephrine								
administration interval	n	dosing	n	combination	n	combination with	setting	n
1 x / day	1	1 mg	21	yes	17	HS	PCU	17
2 x / day	6	2 mg	4	no	14	HS - lidocaine	SAPV	15
3 x / day	9	3 mg	1	NA	1		PCIC	11
4 x / day	2	4 mg	1				NP	7
3 - 4 x / day or PRN	1	FT: 5 mg	1				NA	0
PRN	8	VOR	2					
VOR	5	NA	3					
NA	0							

Tranexamic acid								
administration interval	n	dosing	n	combination	n	combination with	setting	n
1 x / day	2	250 mg	8	yes	3	NA	PCU	16
2 x / day	6	500 mg	15	no	22		SAPV	15
3 x / day	10	VOR	2	NA	2		PCIC	5
4 x / day	1	NA	2				NP	4
PRN	3						NA	0
VOR	4							
NA	1							

Morphine								
administration interval	n	dosing	n	combination	n	combination with	setting	n
2 x / day	1	5 mg	4	yes	4	HS	PCU	1
3 x / day	2	10 mg	1	no	1		SAPV	5
PRN	2	NA	0	NA	0		PCIC	0
NA	0						NP	2
							NA	0

Hydromorphone								
administration interval	n	dosing	n	combination	n	combination with	setting	n
3 x / day	1	1 mg	1	yes	1	NA	PCU	1
NA	0	NA	0	no	0		SAPV	1
				NA	0		PCIC	0
							NP	1
							NA	0

Fentanyl								
administration interval	n	dosing	n	combination	n	combination with	setting	n
1 x / day	1	25 µg	8	yes	3	HS	PCU	10
2 x / day	1	50 µg	7	no	13		SAPV	10
3 x / day	2	FT: 100 µg	1	NA	0		PCIC	3
4 x / day	2	VOR	3				NP	4
bis 6 x / day	1	NA	0				NA	0
PRN	6							
VOR	3							
NA	0							

Ketamine								
administration interval	n	dosing	n	combination	n	combination with	setting	n
2 x / day	1	25 mg	5	yes	1	HS	PCU	4
3 x / day	1	50 mg	1	no	5		SAPV	4
up to 4x / day	1	NA	0	NA	0		PCIC	0
4 x / day	1						NP	0
PRN	1						NA	0
VOR	1							
NA	0							

Lidocaine								
administration interval	n	dosing	n	combination	n	combination with	setting	n
1 x / day	2	10 mg	6	yes	7	HS	PCU	10
2 x / day	3	20 mg	7	no	9		SAPV	9
3 x / day	6	50 mg	3	NA	1		PCIC	3
4 x / day	4	VOR	1				NP	3
PRN	2	NA	0				NA	0
NA	0							

Iloprost								
administration interval	n	dosing	n	combination	n	combination with	setting	n
4 - 6 x / day	1	2.5 µg	1	yes	0	NA	PCU	0
NA	0	NA	0	no	1		SAPV	0
				NA	0		PCIC	0
							NP	1
							NA	0

Tobramycin								
administration interval	n	dosing	n	combination	n	combination with	setting	n
2 x / day	4	80 mg	1	yes	1	HS	PCU	4
NA	0	160 mg	1	no	3		SAPV	1
		300 mg	1	NA	0		PCIC	3
		NA	1				NP	2
							NA	0

Gentamicin								
administration interval	n	dosing	n	combination	n	combination with	setting	n
2 x / day	4	80 mg	4	yes	1	HS	PCU	3
NA	0	NA	0	no	3		SAPV	1
				NA	0		PCIC	4
							NP	3
							NA	0

Colistin								
administration interval	n	Dosis	n	combination	n	combination with	setting	n
3 x / day	1	1 m IU	1	yes	0	NA	PCU	1
NA	0	NA	0	no	1		SAPV	1
				NA	0		PCIC	0
							NP	0
							NA	0

Aztreonam								
administration interval		Aztreonam		combination		combination with	setting	
NA		NA		NA		NA	NA	
